# Advancing the Understanding of Vitamin D Status in Post-Thyroidectomy Hypocalcemia

**DOI:** 10.1155/2021/5598319

**Published:** 2021-04-01

**Authors:** Gurdeep Singh, Fatima Irshaidat, Christopher Lau, Ariel Pedoeem, Christine Feng, Maria Mohammed Fariduddin, Lei Lei Min, Nidhi Bansal

**Affiliations:** ^1^Our Lady of Lourdes Memorial Hospital, 161 Riverside Drive, Binghamton 13905, NY, USA; ^2^Upstate University Hospital, 3229 East Genesee Street, Syracuse 13214, NY, USA

## Abstract

**Background:**

Post-thyroidectomy hypocalcemia is the most common complication after total thyroidectomy. Studies to examine the role of low vitamin D in increasing post-thyroidectomy hypocalcemia incidence have produced varying results. This study aimed to assess whether vitamin D deficiency increases the risk of post-thyroidectomy hypocalcemia.

**Methods:**

This retrospective study involved 244 patients who underwent total thyroidectomy between 2014 and 2019. Patients were divided into two groups based on pre-operative vitamin D levels. Group A and Group B had pre-operative vitamin D (25-hydroxyvitamin D) levels of ≥20 ng/ml and <20 ng/ml (reference range for vitamin D is 30–100 ng/dl). The effect of vitamin D, gender, body mass index (BMI), and ethnicity on post-operative calcium and PTH levels was analyzed.

**Results:**

Post-operative calcium levels for Group A were not statistically different compared to Group B (8.52 ± 0.64 mg/dl vs. 8.45 ± 0.58 mg/dl (mean ± S.D.; *p* value = 0.352). The average post-operative PTH of the two groups did not differ significantly (Group A: 32.4 ± 27.5 pg/ml; Group B: 34.4 ± 41.7 pg/ml; *p* value = 0.761).

**Conclusion:**

Pre-operative vitamin D levels are not predictive of post-thyroidectomy hypocalcemia.

## 1. Introduction

Post-thyroidectomy hypocalcemia is the most common complication of thyroidectomy and can cause transient or permanent hypocalcemia [1, 2]. Several studies examined the relationship between vitamin D levels and hypocalcemia, but the results were inconsistent. Some studies showed a relationship between pre-operative vitamin D deficiency and post-thyroidectomy hypocalcemia, whereas others did not [3]. The incidence of thyroid cancer is increasing and vitamin D deficiency is prevalent worldwide [4, 5]. Since the 1970s, thyroid cancer incidence has increased rapidly, although mortality due to thyroid cancer has remained stable [6]. Between 2000 and 2014, 44,537 thyroidectomies were performed to treat thyroid cancer [7]. Hospitals aim to shorten the post-surgical stay for thyroidectomy patients [8], and there is an ongoing trend for these surgeries to be done on an outpatient basis [9].

Based on these factors, determining whether vitamin D levels should be normalized before total thyroidectomy is essential to reduce the risk of morbidity due to symptomatic hypocalcemia and associated prolonged hospital stays.

## 2. Materials and Methods

This was a retrospective study of 244 patients who underwent total thyroidectomy at our institute, Our Lady of Lourdes Memorial Hospital (Binghamton, New York), between 2014 and 2019, and at Upstate University Hospital (Syracuse, New York), between 2014 and 2018.

The study protocol was approved by Our Lady of Lourdes Memorial Lourdes Hospital IRB; informed consent was not obtained due to the retrospective nature of the study.

None of these patients received prophylactic calcitriol or calcium before thyroidectomy. Laboratory results, including levels of calcium and PTH, were obtained within two weeks after thyroidectomy. Post-operative hypocalcemia was defined as a calcium level below the normal laboratory reference range.

### 2.1. Inclusion Criteria

All patients who underwent total thyroidectomy and had normal pre-operative calcium and vitamin D levels within six months of surgery were included.

### 2.2. Exclusion Criteria

Patients who underwent hemithyroidectomy, central or lateral neck dissection, and parathyroidectomy, as well as those who were taking bisphosphonates, were excluded.

## 3. Results

A total of 244 patients that underwent total thyroidectomy between 2014 and 2019 were considered. Of these, 151 had vitamin D (25-hydroxyvitamin D) levels ≥20 ng/ml (Group A), and the remaining 93 patients had low levels <20 ng/ml (Group B). The reference range for 25-hydroxyvitamin D was 30–100 ng/ml.

The demographics and biochemical profiles for both groups are presented in Table 1. The average age of patients in Group A was higher than that of Group B (49.4 years old vs. 45.5 years old), and both groups had more females than males (84.8% and 78.5% for Group A and Group B, respectively). The percentage of white patients was 92% and 75.3% for Group A and Group, respectively.

Patients in both Group A and Group B were more likely to have benign than cancerous thyroid pathology (Group A: 65.6% (99) vs. 34.4% (52); Group B: 60.2% (56) vs. 39.8% (37)). Papillary thyroid cancer was present in 90.4% of cancerous cases in Group A, while 9.6% had follicular cancer. For Group B, the percentage of papillary thyroid cancer was lower at 78.4% and follicular cancer was higher at 21.6%.

There was no significant difference between the two groups for the average pre-operative calcium level (Group A: 9.15 ± 0.58 mg/dl and Group B: 9.06 ± 0.48). One-way analysis of variance (ANOVA) carried out using Minitab v.18 also showed no significant difference in the level of post-operative calcium for the two groups (Group A: 8.52 ± 0.64 mg/dl and Group B: 8.45 ± 0.58 mg/dl; *p*=0.352). These results indicated no correlation between pre-operative vitamin D deficiency and post-thyroidectomy hypocalcemia.

**Figure 1:**
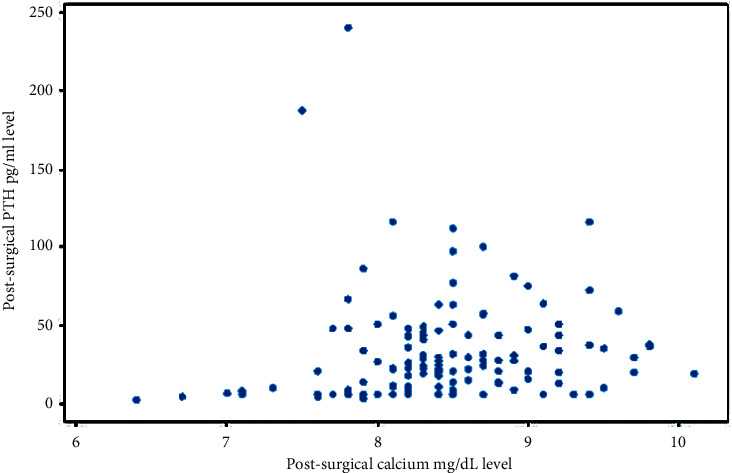
Pearson correlation coefficient between post-surgical calcium and post-surgical parathyroid hormone was 0.102 indicating a weak correlation.

Post-operative PTH was available for 143 (58.4%) of the patients in this study. The average level of post-operative PTH for the two groups was similar (Group A, 32.4 ± 27.5 pg/mL (*n* = 88) vs. Group B, 34.4 ± 41.7 pg/ml (*n* = 55); *p*=0.761; Table 2). Overall, the correlation between post-surgical calcium and post-surgical PTH levels was weak (Pearson correlation coefficient = 0.102; *p* value = 0.223; Figure 1).

We further divided the patient cohort such that Group A had vitamin D levels ≥20 ng/ml, Group B levels were ≥10 ng/ml and <20 ng/ml, and Group C had levels <10 (Table 3). Groups A, B, and C had 151, 79, and 14 patients, respectively. The post-operative calcium levels were similar between the groups (8.52 ± 0.64 mg/dl, 8.46 ± 0.6 mg/dl, and 8.36 ± 0.46 mg/dl, Groups A, B, and C, respectively; *p*=0.448), indicating no difference in the incidence of hypocalcemia after thyroidectomy among the groups.

Patients with BMI <30 tended to have significantly higher post-operative PTH values than patients with BMI >30 (40.13 pg/ml vs. 27.52 pg/ml, *p*=0.034), but the incidence of post-operative hypocalcemia was not significantly affected by body weight (*p*=0.477).

Post-operative PTH levels among different ethnic groups varied significantly in this study cohort (*p*=0.029). On average, African Americans tended to have the highest post-operative PTH levels (10 patients, mean value = 45.8 pg/ml; 95% CI 4.4, 87.2). The 126 patients with ethnicity listed as “White” had a mean value of 33.12 pg/ml (CI 27.55, 38.70) and the ethnic group listed as other (*n* = 7) had the lowest mean value of 15.83 pg/ml. Although the “White” group tended to have the highest post-operative calcium levels, followed by African Americans and “other,” the differences among these groups were not significant (*p*=0.099).

Gender also had no significant effect on post-operative PTH level (*p*=0.185) and post-operative hypocalcemia (*p*=0.997).

## 4. Discussion

Hypocalcemia is a well-known post-operative complication of thyroidectomy, but it is primarily transient (less than six months). A meta-analysis by Edafe et al. reported that the incidence of transient and permanent hypocalcemia was 27% (19%–38%) and 1% (0%–3%), respectively [10].

Post-thyroidectomy hypocalcemia can cause significant morbidity and lengthen hospital stays. Multiple factors can increase the risk of post-surgical hypocalcemia, including autoimmune thyroid disease, substernal goiter, central neck dissection, simultaneous thyroidectomy and parathyroidectomy, prior gastric bypass surgery, previous central neck surgery, and low volume thyroid surgeon [11]. However, whether vitamin D is predictive of hypocalcemia risk is unclear.

Multiple studies evaluated the role of vitamin D in post-thyroidectomy hypocalcemia. However, the inclusion criteria, including the definition of vitamin D deficiency, the extent of thyroid surgery, and the inclusion of parathyroidectomy or neck dissection, differed among the studies and, in turn, could have produced different outcomes.

Multiple studies reported that vitamin D levels <20 ng/ml did not increase the risk of hypocalcemia after total thyroidectomy [12–14]. Meanwhile, Salinger et al. used a vitamin D level <30 ng/ml, and both Godazandeh et al. and Kim et al. used a vitamin D level <10 ng/ml. However, despite these different threshold values, none of these studies found vitamin D levels to be predictive of the risk of hypocalcemia after thyroidectomy [15–17].

In a retrospective study on 213 patients who underwent total or completion thyroidectomy, Al-Khatib et al. found that a vitamin D level of 25 nmol/L was predictive of post-operative hypocalcemia [18]. The more extensive meta-analysis by Edafe et al. found similar results [10].

Our study analyzed the data using vitamin D thresholds of ≥20 ng/ml and ≤20 ng/ml to group the patients. Consistent with some earlier studies, we also found no relationship between vitamin D deficiency and post-thyroidectomy hypocalcemia. Considering a lower threshold of <10 ng/ml also showed no relationship with the risk of post-thyroidectomy hypocalcemia.

Our study's results align with those reported by Griffin et al., Salinger et al., Godazandeh et al., and Cherian et al., but the sample size was <150 in these studies compared to 244 in our study. Another strength of our study is that more than one surgeon performed total thyroidectomies.

Our study's limitations are its retrospective nature and the availability of post-operative PTH values for just over half (143/244; 58.4%) of the patients. Another limitation is that we evaluated the calcium and PTH within 2 weeks after total thyroidectomy only, so this study does not differentiate between temporary or permanent hypoparathyroidism.

A significant body of data indicates that prophylactic use of calcium or vitamin D can decrease the risk of post-operative hypocalcemia [19]. In a review of 15 studies, Gregory et al. found that perioperative vitamin D and calcium supplements effectively prevented both laboratory and symptomatic hypocalcemia [20]. Furthermore, a meta-analysis by Xing et al. reported that post-operative use of calcium and vitamin D had a better preventative effect than calcium alone [21].

In the present study, we found that higher BMI (>30) could be related to a significantly lower PTH level but saw no relationship between BMI and post-operative hypocalcemia. A prospective study by Final et al. reported a lower incidence of permanent hypoparathyroidism (1.05 vs. 1.38%) in patients with BMI <25 compared to those with BMI ≥25, but this difference was not statistically significant [22].

Moreover, ethnicity did seem to affect the post-operative level of PTH, but not post-operative calcium, although a larger sample size is needed to confirm this relationship.

Perioperative supplements with vitamin D and calcium appear to prevent symptomatic hypercalcemia effectively. However, there is insufficient evidence to indicate that pre-operative vitamin D deficiency is a risk factor for post-operative hypocalcemia.

## 5. Conclusion

Vitamin D levels were not predictive of post-thyroidectomy hypocalcemia at our institution. Correcting vitamin D levels before total thyroidectomy to reduce the risk of morbidity due to symptomatic hypocalcemia and prolonged hospital stays is unnecessary. Previous studies on the association of vitamin D with post-operative hypocalcemia have produced varying results. These differences could be due to their retrospective nature and involvement of only one center and the multiple risk factors that contribute to the risk of hypocalcemia. Consideration of all these factors in retrospective studies can be challenging. Thus, a multicenter, randomized control trial is needed to determine whether pre-operative normalization of vitamin D should be recommended before total thyroidectomy.

## Figures and Tables

**Table 1:** Patient characteristics.

	Group A, Vit-D> = 20 ng/ml mean (*N*) ± SD	Group B, Vit-D <20 ng/ml	*p* value
Age	49.4 (138) ± 17.3	45.5 (86) ± 14.4	0.072

Gender			0.363
Male	15.2% (20)	21.5% (20)	
Female	84.8% (128)	78.5% (73)	

Ethnicity			0.003
White	92% (139)	75.3% (70)	
African American	4% (6)	16.1% (15)	
Other	4% (6)	8.6% (8)	

BMI	31 (150) ± 6.97	33.23 (92) ± 9.04	0.044
Thyroid pathology			0.553
Percentage (*N*)			
Cancerous	34.4% (52)	39.8% (37)	
Benign	65.6% (99)	60.2% (56)	

Thyroid cancer			0.076
Percentage (*N*)			
Papillary	90.4% (47)	78.4% (29)	
Follicular	9.6% (5)	21.6% (8)	

Vitamin D ng/ml	30.85 (151) ± 9.48	13.47 (93) ± 4.3	Not applicable
Pre-op calcium mg/dl	9.15 (151) ± 0.58	9.06 (93) ± 0.48	0.168
Post-op calcium mg/dl	8.52 (151) ± 0.64	8.45 (93) ± 0.58	0.352
Post-op PTH pg/ml	32.4 (88) ± 27.5	34.4 (55) ± 41.7	0.761

**Table 2:** Relationship of post-surgical PTH and Ca levels with potential risk factors for post-operative hypocalcemia.

Factor; level			Post-surgical PTH (pg/ml)	Post-surgical Ca (mg/dl)
Pre-surgical vitamin D ng/ml	*p* value		0.761	0.352
	*D* <20 ng/ml	Mean (*N*) (confidence interval)	34.35 (55) (23.08, 45.63)	8.45 (93) (8.3256, 8.5647)
	*D*> = 20 ng/ml	Mean (*N*) (confidence interval)	32.42 (88) (26.58, 38.25)	8.52 (151) (8.4168, 8.6216)

Ethnicity	*p* value		0.029	0.099
	African American	Mean (*N*) (confidence interval)	45.8 (10) (4.4, 87.2)	8.45 (21) (8.109, 8.786)
	Other	Mean (*N*) (confidence interval)	15.83 (7) (3.57, 28.09)	8.08 (14) (7.665, 8.492)
	White	Mean (*N*) (confidence interval)	33.12 (126) (27.55, 38.70)	8.52 (209) (8.4428, 8.6031)

Gender	*p* value		0.185	0.997
	Female	Mean (*N*) (confidence interval)	30.74 (121) (25.62, 35.87)	8.45 (201) (8.4040, 8.5780)
	Male	Mean (*N*) (confidence interval)	46.5 (22) (23.1, 69.8)	8.49 (43) (8.3134, 8.6680)

BMI	*p* value		0.034	0.477
	Normal BMI <30	Mean (*N*) (confidence interval)	40.13 (64) (29.73, 50.54)	8.46 (109) (8.3394, 8.5824)
	High BMI > = 30	Mean (*N*) (confidence interval)	27.52 (79) (22.13, 32.90)	8.52 (133) (8.4159, 8.6202)

**Table 3:** Relationship between pre-operative vitamin D levels and post-operative hypocalcemia and PTH.

	Group A, Vit-D > = 20 ng/ml	Group B, Vit-D <20 ng/ml	Group C, Vit-D< 10 ng/ml	*p* value
Post-operative hypocalcemia	8.52 ± 0.64 mg/dl (151)	8.46 ± 0.6 mg/dl (79)	8.36 ± 0.46 mg/dl (14)	0.448
Post-operative PTH	32.42 ± 27.53 pg/ml (88)	25.47 ± 19.4 pg/ml (46)	79.8 ± 82.8 pg/ml (9)	0.07
Age (yr)	49.7 ± 16.9 (138)	45.4 ± 14.1 (73)	46.3 ± 16.7 (13)	

## Data Availability

The dataset generated during the current study is available from the corresponding author on reasonable request.
